# Multi-omic analyses reveal the waterlogging induced responses in *Magnolia sinostellata*

**DOI:** 10.3389/fpls.2025.1653464

**Published:** 2025-09-11

**Authors:** Xiaoai Fang, Lu Fan, Huijuan Zhou, Huiling Yan, Fangbing Ding, Renna Li, Yuwei Linghu, Bin Xie, Yaling Wang

**Affiliations:** ^1^ Xi’an Botanical Garden of Shaanxi Province, Institute of Botany of Shaanxi Province, Xi’an, China; ^2^ College of Tropical Agriculture and Forestry, Hainan University, Haikou, China

**Keywords:** waterlogging stress, *Magnolia sinostellata*, transcriptomic, metabolomic, plant signal transduction pathway

## Abstract

Waterlogging stress poses a significant constraint on the cultivation and landscape utilization of Magnolia species. Currently, the molecular mechanisms underlying their adaptation remain largely unexplored. *Magnolia sinostellata*, a riparian species with exceptional waterlogging tolerance, provides an ideal model to decipher these mechanisms. Here, we integrated transcriptomic and metabolomic analyses to investigate the dynamic responses of different tissues (roots, stems, leaves) in *M. sinostellata* to waterlogging stress at 0 h, 6 h, and 72 h. Roots showed the strongest response, with 12,538 DEGs and 178 DEMs. Additionally, the morphological adaptations included hypertrophic lenticel, aerenchyma formation and adventitious root development. The combined analysis of transcriptome and metabolome indicates that the plant signal transduction pathway plays an important role in responding to waterlogging stress. Our findings demonstrate that multiple phytohormone signaling pathways, including IAA, JA, CTK, GA, and ET, collectively regulate the tolerance of *M. Sinostellata to* waterlogging stress. Notably, we identified jasmonic acid (JA) as a negative regulator of this adaptive response, contrasting with its positive role in other species, and pinpointed key candidate genes (*CKX* and *JAR1*). Taken together, this study advances our theoretical understanding of woody plant adaptation to waterlogging stress and delivers practical genetic tools for developing waterlogging-resistant ornamental cultivars.

## 1 Introduction

Waterlogging has emerged as a critical environmental constraint that severely impairs plant growth, distribution, and productivity across natural ecosystems by disrupting water balance, altering morphology, and suppressing metabolic activity (Xu et al., 2024; Bidalia et al., 2018). Climate change results in the increasing incidence of waterlogging events (Li et al., 2022). Therefore, researchers are conducting extensive studies to unravel the mechanisms behind plant hypoxia response, aiming to develop waterlogging-resistant varieties (Liu et al., 2023; Shi et al., 2024). Waterlogging triggers hypoxia-induced physiological and biochemical alterations, characterized by increased redox potential and reactive oxygen species (ROS) accumulation, ultimately resulting in oxidative damage and membrane lipid peroxidation (Xu et al., 2022; Arbona et al., 2017; Gang et al., 2020). To mitigate these effects, plants employ integrated adaptive strategies spanning multiple organizational levels: morphological adaptations (adventitious root formation and aerenchyma development) (Bailey-Serres and Voesenek, 2008; Dawood et al., 2016), metabolic reprogramming (anaerobic respiration and stress-induced metabolic shifts) (Voesenek and Bailey-Serres, 2015), and dynamic transcriptional regulation of stress-responsive genes (Zhang et al., 2016). With advancements in high-throughput RNA sequencing (RNA-seq) technology, numerous studies have employed transcriptomic approaches to dissect the molecular response mechanisms in crops, forestry species, and fruit trees (Wang et al., 2024; Zhang et al., 2023, 2024, 2025). These studies have provided valuable insights into the molecular basis of waterlogging tolerance. However, woody ornamentals lack equivalent mechanistic studies, particularly regarding phytohormone networks that integrate stress signaling with developmental plasticity.

Plant hormones serve as critical chemical messengers that coordinate both developmental programs and adaptive responses to waterlogging stress across all growth stages (Fukao et al., 2019; Huang et al., 2022). These signaling molecules, including ethylene (ET), auxin (IAA), abscisic acid (ABA), gibberellin (GA), and jasmonate acid (JA), form an intricate network that modulates physiological and morphological adaptations to oxygen deprivation (Voesenek and Bailey-Serres, 2015). Under waterlogging stress, they undergo dramatic reprogramming to initiate survival strategies, making them central to plant resilience in waterlogging-prone environments (Sasidharan et al., 2018). The rapid accumulation of ET serves as the primary hypoxia signal, triggering a cascade of downstream responses (Hartman et al., 2019). Adventitious root development progresses are differentially regulated through complex interactions between ET, GA, and ABA signaling cascades (Steffens et al., 2006). IAA regulates the formation of adventitious roots mainly through its transport and signal transduction pathways (Pamfil and Bellini, 2011; Adem et al., 2024). Recent studies have revealed that JA plays a dual role, both during the hypoxia phase and the critical reoxygenation period following waterlogging. Meanwhile, the regulation of waterlogging stress by JA shows differences among different species (Yuan et al., 2017; Pan et al., 2025). These hormonal interactions are further fine-tuned by ROS signaling, creating a complex but highly coordinated defense network (Bailey-Serres and Voesenek, 2008). As climate change increases the frequency of extreme rainfall events, elucidating the precise roles of phytohormones in waterlogging responses becomes increasingly crucial for the application of horticultural ornamental plants (Sasidharan et al., 2018). Understanding these hormonal mechanisms has significant implications for developing waterlogging-resistant varieties through both conventional breeding and biotechnological approaches (Septiningsih and Mackill, 2018). The identification of key regulatory genes in hormone pathways offers promising targets for genetic improvement of waterlogging tolerance (Loreti et al., 2016).

*Magnolia* species possess both ornamental, medicinal, timber, ecological and research values, and are important plant materials for building low-carbon ecological gardens. However, due to their mostly fleshy roots and their aversion to low humidity, when the planting area is flooded, the roots are prone to rot. This characteristic greatly limits their application in garden green spaces, especially in regions similar to East China and South China where there is frequent rainfall and seasonal soil waterlogging (Wang et al., 2022). In contrast to most magnolias, *M. sinostellata* thrives in riparian habitats, a trait rarely observed in the genus (Zhang, 2013; Yu et al., 2019; Wang et al., 2022). This rare intraspecific variation makes it an ideal model for analyzing the waterlogging adaptation mechanism of woody plants. We hypothesized that *M. sinostellata* employs unique physiological and molecular adaptations, distinct from other *Magnolia* species, to cope with waterlogging stress. Recent advances in waterlogging-tolerant woody plants (e.g., *Malus domestica* and *Prunus persica*) highlight the role of hormone signal transduction (Zhang et al., 2023; Ateeq et al., 2025), but whether *M. sinostellata* employs similar or distinct strategies remains unexplored. To address this knowledge gap, our investigation employed *M. sinostellata* as a model system to systematically decode its hydraulic adaptation mechanisms through an integrated transcriptomic-metabolomic framework. This study has enhanced our understanding of the response of *M. sinostellata* to waterlogging stress, revealing the potential regulatory pathways and candidate genes involved in this process. It provides theoretical support for molecular breeding of the purple flower magnolia for waterlogging tolerance. Meanwhile, it explores the molecular mechanism of waterlogging tolerance in the purple flower magnolia, providing molecular markers for the selection of new varieties of water-tolerant magnolia, which is helpful in addressing the breeding bottlenecks in current urban greening.

## 2 Materials and methods

### 2.1 Plant materials and waterlogging stress treatment

The one-year-old cutting seedlings of *M. sinostellata* were obtained from Xi’an Botanical Garden, Shaanxi Province, China. Uniform plants were acclimatized for 7 days in a greenhouse (25 ± 1 °C, 60% relative humidity, 14 h light/10 h dark cycle, 300 μmol m^−2^ s^−1^ photosynthetically active radiation (PAR)) prior to experiments. Waterlogging stress was imposed by placing potted plants in a water tank (80 cm × 57 cm × 30 cm) filled with dechlorinated tap water (pH 6.5 ± 0.2, dissolved oxygen: 2.8 ± 0.3 mg L^−1^ at 25 °C) to 10 cm above the soil surface. The leaves, stems and roots of *M. sinostellata* were collected at 0 h, 6 h, and 72 h post waterlogging stress, with 3 biological replicates conducted at each time point. The morphological changes were observed.

### 2.2 Measurement of morphological and anatomical observations

The roots of *M. sinostellata* were washed with deionized water, and root morphology was photographed by Epson Perfection V700 Photo (Epson Co., Ltd., China). Root tips were cut by a knife blade, fixed in formalin-acetic acid-alcohol (FAA), and then conducted by using a Saffron-O and Fast Green Stain Kit (Solarbio, Beijing, China) based on the instructions of manufacturers. The cell morphology of root tip was viewed with an optical microscope (BX43, Olympus, Tokyo, Japan).

### 2.3 Transcriptome sequencing and data analysis

The total RNA of *M. sinostellata* was extracted using Trizol reagent (Invitrogen) and quality was verified by agarose gel electrophoresis, NanoPhotometer spectrophotometry (Implen), and Bioanalyzer 2100 (Agilent Technologies). Qualified RNA samples were sequenced on the Illumina HiSeq-2000 platform (Wuhan Maiwei Metabolic Biotechnology Co.). Raw reads were processed using Trimmomatic v0.33 to remove adapter sequences and low-quality reads (>10% N or >50% bases with Q ≤ 20). Clean reads were aligned to the *Magnolia biondii* reference genome (https://doi.org/10.5061/dryad.s4mw6m947) using HISAT2, achieving >80% mapping efficiency. Gene expression quantification was performed with HTSeq, and differential expression analysis was conducted using DESeq2 (adjusted p-value (padj) < 0.05, |log2 fold change(FC) |>1). To study the accumulation of specific metabolites, principal component analysis (PCA) and orthogonal partial least squares discriminant analysis (OPLS-DA) were performed using R (www.r-project.org/2). The gene ontology (GO function) analysis of differentially expressed genes was performed by GOseq, including GO function enrichment and GO function clustering of differentially expressed genes. The database used was the gene ontology database (http://www.geneontology.org/). The kyoto encyclopedia of genes and genomes (KEGG) enrichment analysis of differentially expressed genes and differential metabolites was performed using the KOBAS software and KEGG database (http://www.kegg.jp/kegg/pathway.html).

### 2.4 Metabolomic analysis

Samples for metabolomic analysis were collected from the same biological replicates used for transcriptomics, which were snap-frozen in liquid nitrogen and analyzed in triplicate. Untargeted metabolomic profiling was performed using an ultra-performance liquid chromatography (UPLC) system (Shim-pack UFLC SHIMADZU CBM30A, Japan) coupled with a tandem mass spectrometer (QTRAP^®^ 4500, Applied Biosystems, USA). Analytical procedures and data processing followed established methods (Chen et al., 2013). PCA and OPLS-DA were performed using R software to examine metabolic profiles. The relative importance of each metabolite to the OPLS-DA model was evaluated using the variable importance in projection (VIP) scores. Student’s t test was used to test the significance of the expression of each metabolite in each comparison group, and a fold change ≥2 or ≤0.5 and P-value<0.05 were used as the standards for screening for differentially expressed metabolites (DEMs). Identified metabolites were annotated and mapped to KEGG pathway database (http://www.kegg.jp/kegg/pathway.html). The content of phytohormones at different waterlogging stress times of three tissues were determined by MetWare (http://www.metware.cn/).

### 2.5 Integrated transcriptomics and metabolomics analyses

Differentially expressed genes among root developmental stages were used to construct a gene co-expression network with the weighted gene co-expression network analysis (WGCNA) package, which is a representative algorithm used for developing co-expression networks. The soft-thresholding power (β = 18) was selected based on scale-free topology criterion (R² > 0.85) to ensure a biologically meaningful network. A relatively large minimum module size (30) and a medium sensitivity (deepSplit = 2) to cluster splitting were also selected. In the co-expression network, genes were represented by nodes, and the correlation value (weight) between two genes was calculated as the Pearson’s correlation coefficient. Genes in the same module were first visualized with the Cytoscape program. The final networks were designed with the igraph and ggplot2 packages.

### 2.6 qRT-PCR analysis

Five candidate hormone-related genes (cytokinin dehydrogenase MBI06_g28671_MAGBIO and MBI13_g26146_MAGBIO, and jasmonic acid-amino synthetase MBI07_g47338_MAGBIO, MBI07_g46827_MAGBIO, and MBI06_g08192_MAGBIO) were selected based on their significant differential expression in transcriptome analysis and their involvement in hormone signaling pathways linked to waterlogging tolerance. These genes were validated using quantitative real-time polymerase chain reaction (qRT-PCR). Gene-specific primers (**Supplementary Table S1**) were designed for amplification, and reactions were carried out following established protocols (Shu et al., 2018). The housekeeping gene Actin was employed as normalization, and the relative gene expression was calculated using the 2^−ΔΔCt^ method (Livak and Schmittgen, 2001).

### 2.7 Statistical analysis

All quantitative data are presented as mean ± standard deviation (SD). Statistical significance was assessed using one-way analysis of variance (ANOVA) implemented in SPSS software (version 22.0; IBM Corp., Armonk, NY, USA). Data visualization was performed using multiple analytical tools: OriginPro 2022 (OriginLab Corporation, Northampton, MA, USA) for comprehensive graphical representations, Cytoscape (version 3.9.1) for network analyses, and the MetWare Cloud Platform (https://cloud.metware.cn) for specialized bioinformatics visualizations.

## 3 Results

### 3.1 Physiological changes under waterlogging

**Figure 1:**
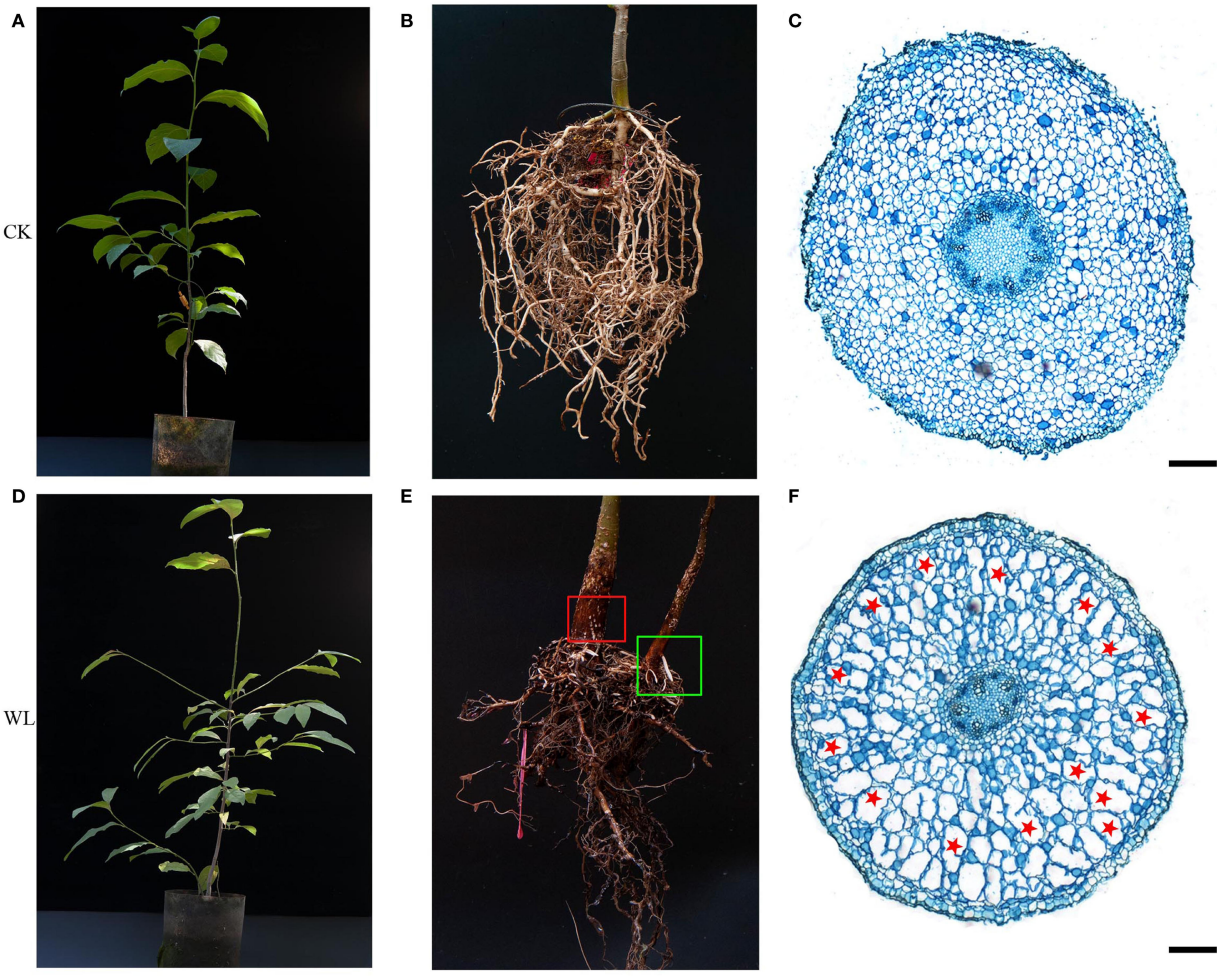
Phenotypic analysis and anatomical analysis of *M. sinostellata*. **(A, D)** Phenotypic analysis of *M. sinostellata.* **(B, E)** Morphological characteristics of roots in *M. sinostellata* at different times (0 h and 72 h) by the waterlogging stress. **(C, F)** Images of stained cross-section of *M. sinostellata* roots under normal and waterlogging conditions for 72 h, bar=50 μm. Boxs indicate hypertrophic lenticels (red) and adventitious roots (green), red stars indicate aerenchymas.

At 72 h of waterlogging compared with the control, *M. sinostellata* displayed green leaves and no signs of damage (**Figures 1A, D**). In the control group, the fibrous roots were numerous and white (**Figure 1B**). After waterlogging stress, some fibrous roots fell off, and the remaining fibrous roots became brown (**Figure 1E**). Meanwhile, the formation of hypertrophic lenticel (**Figure 1E**, red box) and few adventitious roots (**Figure 1E**, green box) on stems and roots were observed at 72 h of waterlogging stress in *M. sinostellata.* In addition, the changes in the internal structure of magnolia roots were further observed through anatomical means. When the root cortex begins to differentiate, the cortical cells in the root of the control group were tightly packed with minimal cell gaps (**Figure 1C**). In contrast, some places in the root cortex of waterlogging groups begin to show cell gaps about half the size of the cell volume, namely aerenchyma (**Figure 1F**, red stars).

### 3.2 Transcriptome analysis

#### 3.2.1 RNA-seq analysis

**Figure 2:**
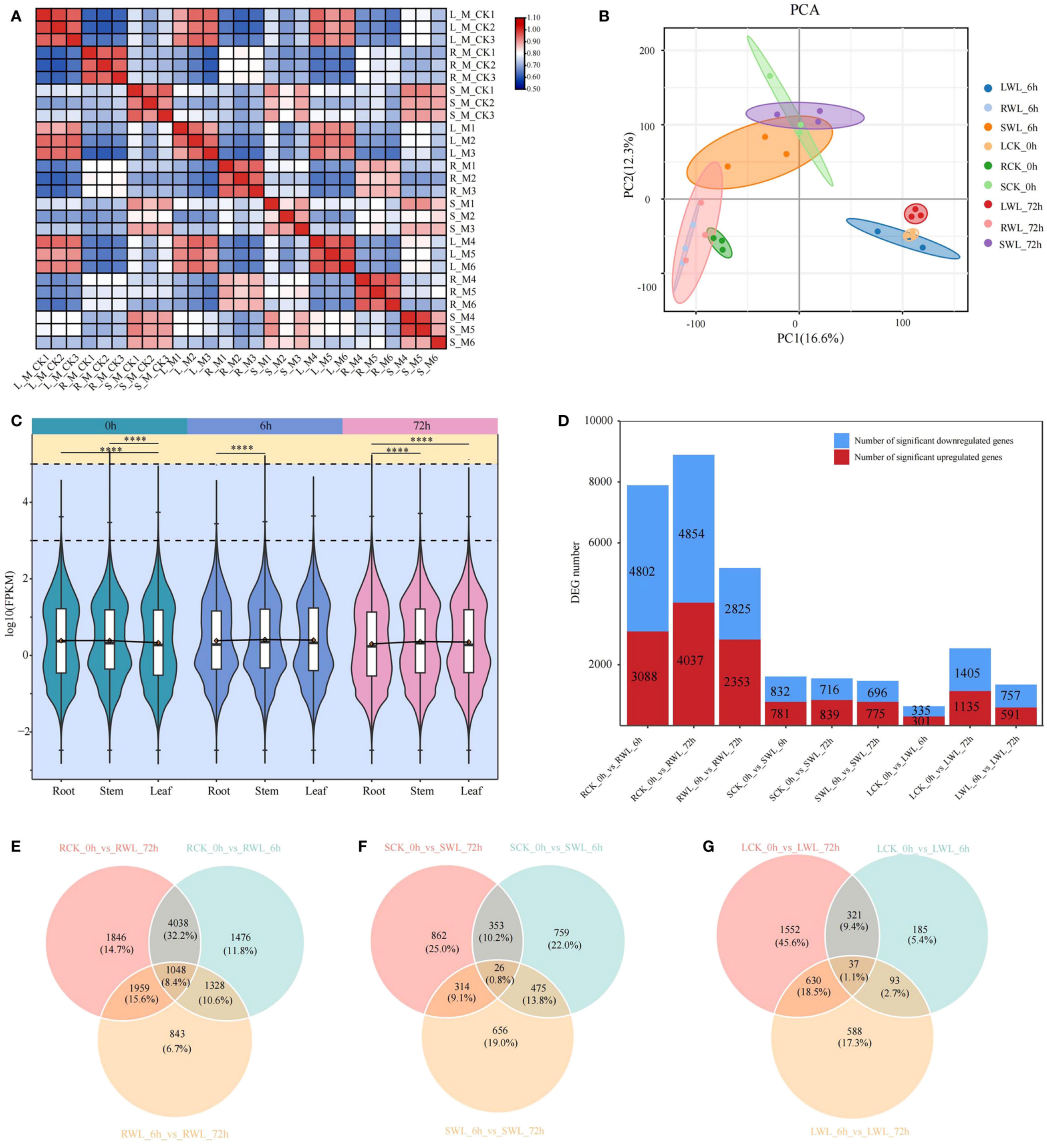
Gene expression analysis of *M. sinostellata* under waterlogging stress. **(A)** Pearson correlation chart shows that the overall sample has a high repeatability. **(B)** PCA diagram. Different colors represent different sample groups. The distance between points on the PCA plot represents how similar all samples are in terms of gene composition and expression. **(C)** Violin plot for differentially expressed genes. (DEGs) expression, different colors represent samples that have undergone waterlogging stress treatment at different times. **(D)** Up-regulation and down-regulation of DEGs. The red boxes represent up-regulation and the blue boxes represent down-regulation. **(E–G)** Co-regulation of DEGs in all comparison groups. ****: P < 0.0001.

To elucidate the molecular mechanisms underlying waterlogging tolerance in *M. sinostellata*, we conducted comprehensive RNA-sequencing analysis of leaf, stem, and root tissues collected at 0 h, 6 h, and 72 h of waterlogging treatments. The sequencing generated 131.25 Gb of raw data, with each sample yielding ≥5.91 Gb of high-quality clean data after filtering (**Supplementary Table S2**). The quality score 20 (Q20) values of each cDNA library were greater than 96.43%, and the average guanine-cytosine content (GC) content was 47.09% (**Supplementary Table S2**). The rate of clean reads mapped to the reference genome of *Magnolia biondii* was higher than 74%. Quality assessment demonstrated excellent experimental reproducibility through strong clustering of biological replicates (intra-group correlation >0.8) (**Figure 2A**), clear separation of control (CK_0h) and waterlogged samples (WL_6h, WL_72h) along PC1, and distinct tissue-specific clustering patterns (roots vs stems vs leaves) along PC2 in PCA analysis (**Figure 2B**). These results demonstrate robust transcriptome profiles suitable for downstream differential expression analysis.

#### 3.2.2 Identification of differentially expressed genes

Using stringent criteria (Padj < 0.05 and |log2(fold change)| > 1), we identified differentially expressed genes (DEGs) across root, stem, and leaf tissues at 0 h (control), 6 h, and 72 h of waterlogging treatment (**Figure 2C**). The analysis revealed substantial tissue-specific responses, with roots exhibiting the most pronounced transcriptional changes (12,538 DEGs), followed by stems (3,445 DEGs) and leaves (3,406 DEGs) (**Figure 2D**). Differential expression analysis demonstrated waterlogging-induced transcriptional changes across tissues (roots > stems > leaves), with roots comparison yielding 7,890 (RCK_0h vs RWL_6h), 8,891 (RCK_0h vs RWL_72h), and 5,178 (RWL_6h vs RWL_72h) DEGs; stems showing 1,613, 1,555, and 1,471 DEGs; and leaves exhibiting 636, 2,540, and 1,348 DEGs in corresponding comparisons. Notably, 1048, 26 and 37 common DEGs were identified in the three tissues under all times of waterlogging stress (**Figures 2E–G**), manifesting that *M. sinostellata* activated the expression levels of these genes to cope with varying times of waterlogging stress. The markedly stronger response in roots underscores their pivotal role in stress perception and initial response. These findings demonstrate that *M. sinostellata* mounts both tissue-specific and shared molecular defenses against waterlogging stress.

#### 3.2.3 Functional enrichment analysis of DEGs

**Figure 3:**
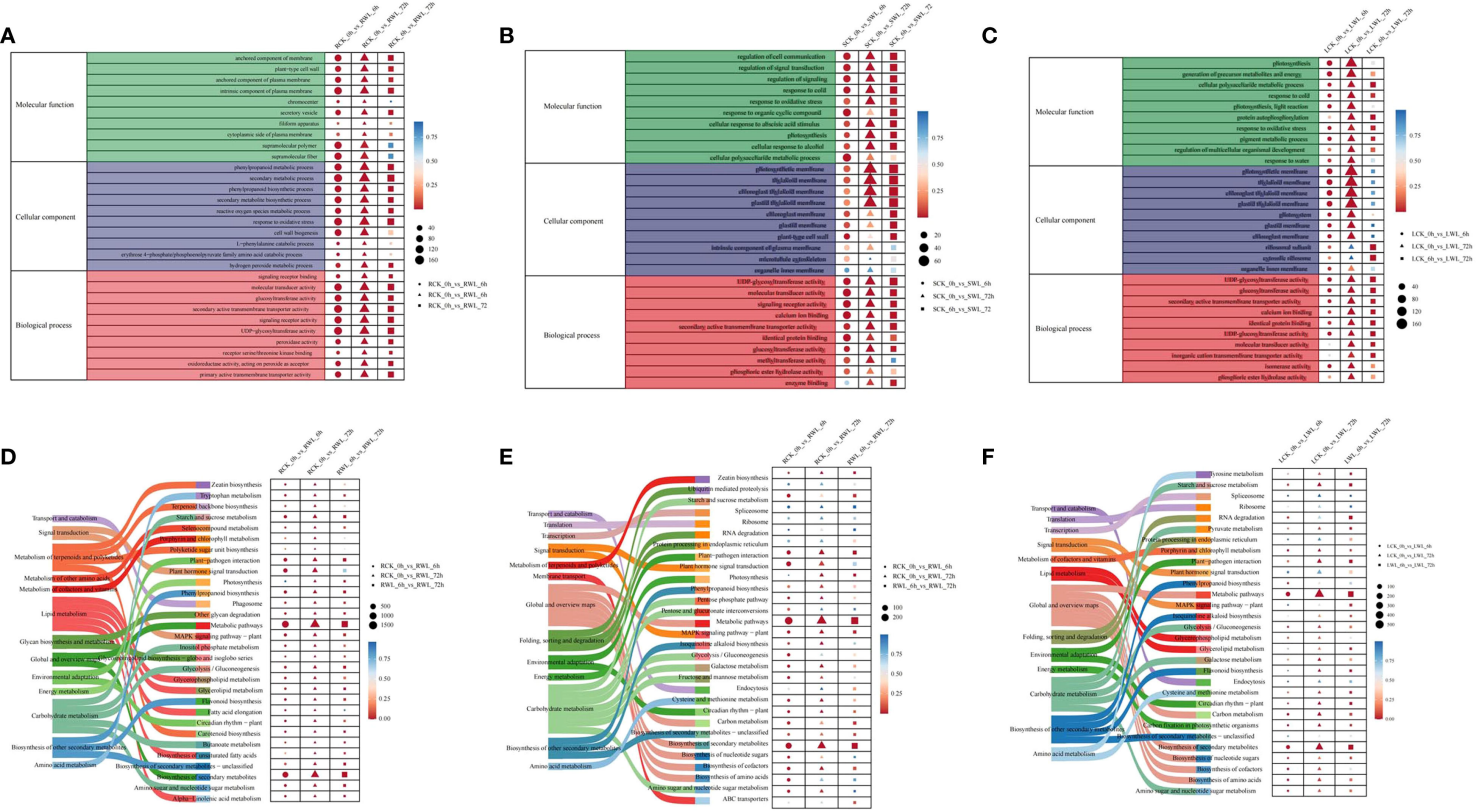
GO and KEGG enrichment analysis of DEGs **(A–C)** GO aggregation and distribution maps shared by the three groups of differential expression genes. The vertical coordinate is the enriched GO term, and the horizontal coordinate is the number and significance of differentially expressed genes in this term. Different colors are used to distinguish biological processes, cellular components, and molecular functions. The colors of the points correspond to different p-value ranges, and different shapes represent different groupings. **(D–F)** KEGG pathway rich distribution map shared by the three groups of differential expression genes. The picture on the left represents the secondary and tertiary pathways of KEGG. In the figure on the right, the vertical axis represents the pathway name, the horizontal axis represents Rich factor, the size of the dots represents the number of differentially expressed genes in this pathway, and the colors of the dots correspond to different p-value ranges, and different shapes represent different groups.

Gene Ontology (GO) enrichment analysis revealed significant organizational differences in biological functions affected by waterlogging stress (**Figure 3A**, **Supplementary Tables S3–S5**). As a result, these DEGs were found to be associated with multiple biological processes, and demonstrated tissue-specific patterns. However, there are also some DEGs that are enriched for the same terms. In the biological process category, the DEGs were predominantly enriched in response to oxidative stress. Likewise, in the cellular component category, these genes exhibited enrichment in plant-type cell wall, intrinsic component of plasma membrane. In the molecular function category, the DEGs were primarily associated with UDP-glycosyltransferase activity、molecular transducer activity, signaling receptor activity, secondary active transmembrane transporter activity, secondary active transmembrane transporter activity. (**Figure 3A**) Notably, the consistent enrichment of oxidative stress response and signal transduction pathways across tissues suggests their central role in the adaptation mechanism of *M. sinostellata to* waterlogging stress.

The KEGG pathway enrichment analysis confirmed the impact of waterlogging stress on specific biological pathways. Various pathways were induced in different tissues of *M. sinostellata* by waterlogging stress. According to the KEGG analysis results (**Supplementary Tables S6–S8**), the pathways of differential gene enrichment in roots, stems and leaves were also similar. Pathways with more gene mappings are metabolic pathways, biosynthesis of secondary metabolites, starch and sucrose biosynthesis, and plant hormone signal transduction (**Figure 3B**). The coordinated induction of these pathways demonstrates the integrated defense strategy of *M. sinostellata*, combining metabolic adjustment, antioxidant production, and hormonal regulation to mitigate waterlogging damage.

### 3.3 Metabolome analysis

#### 3.3.1 Quality control of metabolomic data

**Figure 4:**
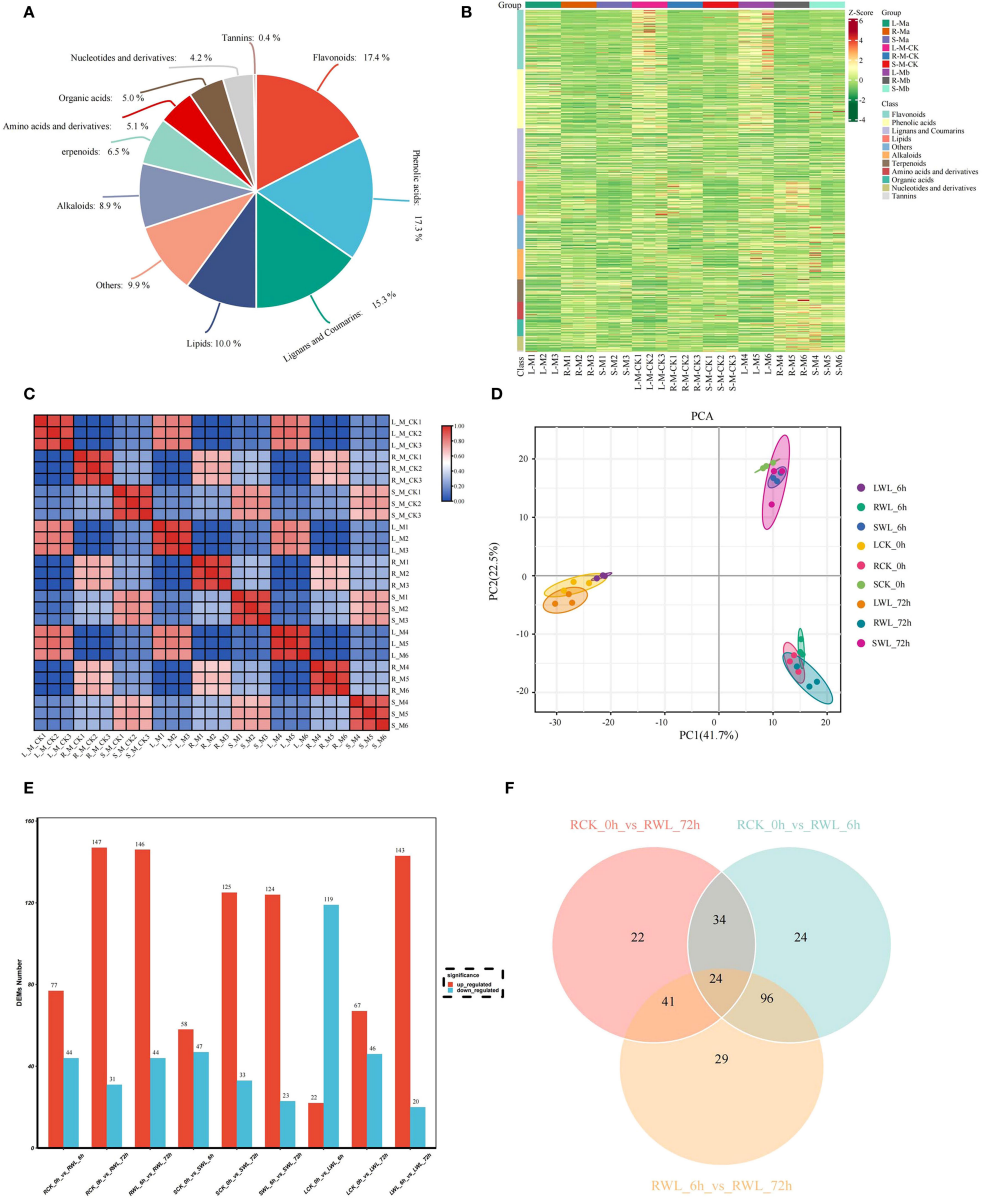
Quality control of metabolomics data and an overview of DEMs identified from *M. sinostellat*a. **(A)** Metabolite classification pie chart, color represents the primary classification of metabolites. **(B)** In the heat map, the horizontal coordinate represents the sample, the vertical coordinate represents the gene, the red is the high expression gene, and the green is the low expression gene. The horizontal comment bar represents the grouping, and the vertical comment bar represents the metabolite classification. **(C, D)** PCA diagram. Different colors represent different groups. The distance between points on the PCA plot represents how similar all samples are in terms of gene composition and expression. **(E)** Differential up-regulated and down-regulated metabolites in different tissues of *M. sinostellata*. **(F)** Venn diagram showing differential metabolites in roots.

Liquid chromatography-quadrupole time-of-flight mass spectrometry (LC-QTOF-MS)-based metabolomic analysis identified 11 major classes of stress-responsive metabolites in *M. sinostellata* (**Figure 4A**, **Supplementary Table S9**). The metabolites were mainly concentrated in Flavonoids (144, 17.4%), Phenolic acids (143, 17.3%), Lignans and Coumarins (127, 15.3%), Lipids (83, 10%), Alkaloids (74, 8.9%), Terpenoids (54, 6.5%), Amino acids and derivatives (42, 5.1%), Organic acids (41, 5.0%), Nucleotides and derivatives (35, 4.2%), Tannins (3, 0.4%) (**Figure 4A**). Multivariate analysis demonstrated significant metabolic reorganization under stress conditions (**Figures 4B, C**). PCA revealed clear separation between control and stressed samples along principal components explaining 64.2% cumulative variance, with tight clustering of biological replicates (R² > 0.85) confirming data reliability (**Figure 4D**). The distinct metabolic signatures observed in different organs (roots > stems > leaves) and time points reflect tissue-specific adaptation strategies to hypoxia.

#### 3.3.2 Differentially expressed metabolites in different parts of *M. sinostellata*

**Figure 5:**
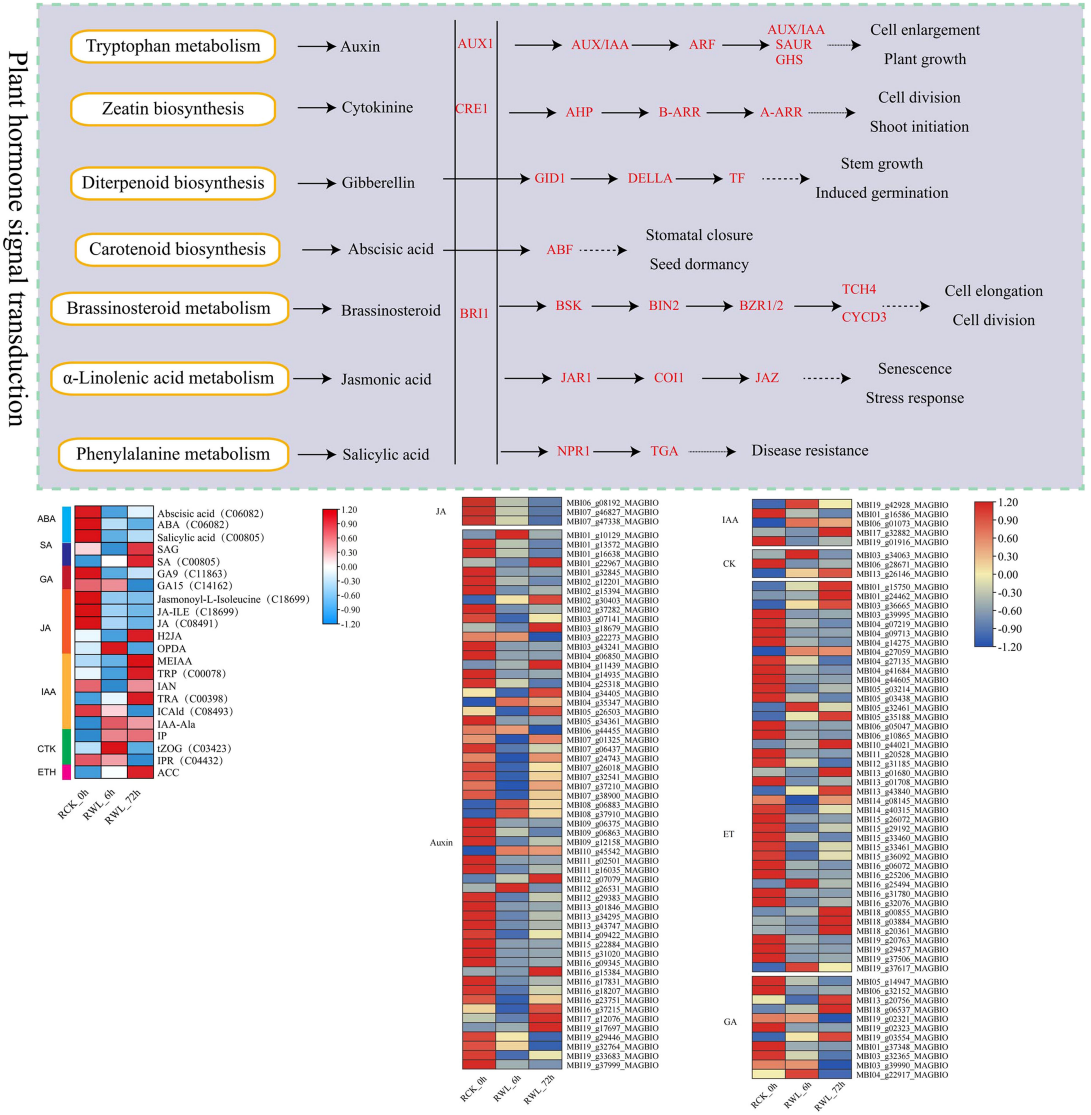
DEGs and DEMs related to plant hormone signaling transduction pathways of *M. sinostellata* in response to waterlogging stress. The samples are displayed below each column. The expressions of the DEGs and DEMs are displayed in different colors. Red means high expression and blue means low expression.

The DEMs were identified according to VIP > 1.0, FC ≥ 2, or FC ≤ 0.5 and P-value < 0.05. By comparing root (R), stem (S) and leaves (L) samples of *M. sinostellata* under different conditions (CK and WL), 121 (77 up-regulated, 44 down-regulated), 178 (147 up-regulated, 31 down-regulated), 190 (146 up-regulated, 44 down-regulated), 105 (58 up-regulated, 47 down-regulated), 158 (125 up-regulated, 33 down-regulated), 147 (124 up-regulated, 23 down-regulated), 141 (22 up-regulated, 119 down-regulated), 113 (67 up-regulated, 46 down-regulated), and 163 (143 up-regulated, 20 down-regulated) DEMs were obtained from RCK_0h vs RWL_6h、RCK_0h vs RWL_72h、RWL_6h vs RWL_72h、SCK_0h vs SWL_6h、SCK_0h vs SWL_72h、SWL_6h vs SWL_72h、LCK_0h vs LWL_6h、LCK_0h vs LWL_72h and LWL_6h vs LWL_72h, respectively (**Figure 4E**). Notably, more DEMs were up-regulated in the roots and stems after waterlogging stress, and with the extension of time, the DEMs in the roots and stems were gradually increased. More metabolites were down-regulated in leaves at 6h, and the number of DEMs first decreased and then increased with the time of waterlogging stress. Since the transcriptome results show that the root was more obviously responded to waterlogging stress, here we mainly focus on the metabolites in the root. The Venn diagram analysis showed that 24 DEMs were found to be affected by different time of waterlogging stress treatment (**Figure 4F**), which can serve as potential candidate markers for the response of *M. sinostellata* to waterlogging stress. Meanwhile, to explore the role of plant hormones in responding to waterlogging stress, we determined the contents of phytohormones at different waterlogging stress times of root in *M. sinostellata.* A total of 22 phytohormone metabolites were detected in annual shoots, including 6 IAAs, 5 JAs, 3 CTKs, 3 SAs, 2 GAs, 2ABAs, 1 ET (**Figure 5**, **Supplementary Table S10**).

### 3.4 WGCNA and validation of hub genes by qRT-PCR

**Figure 6:**
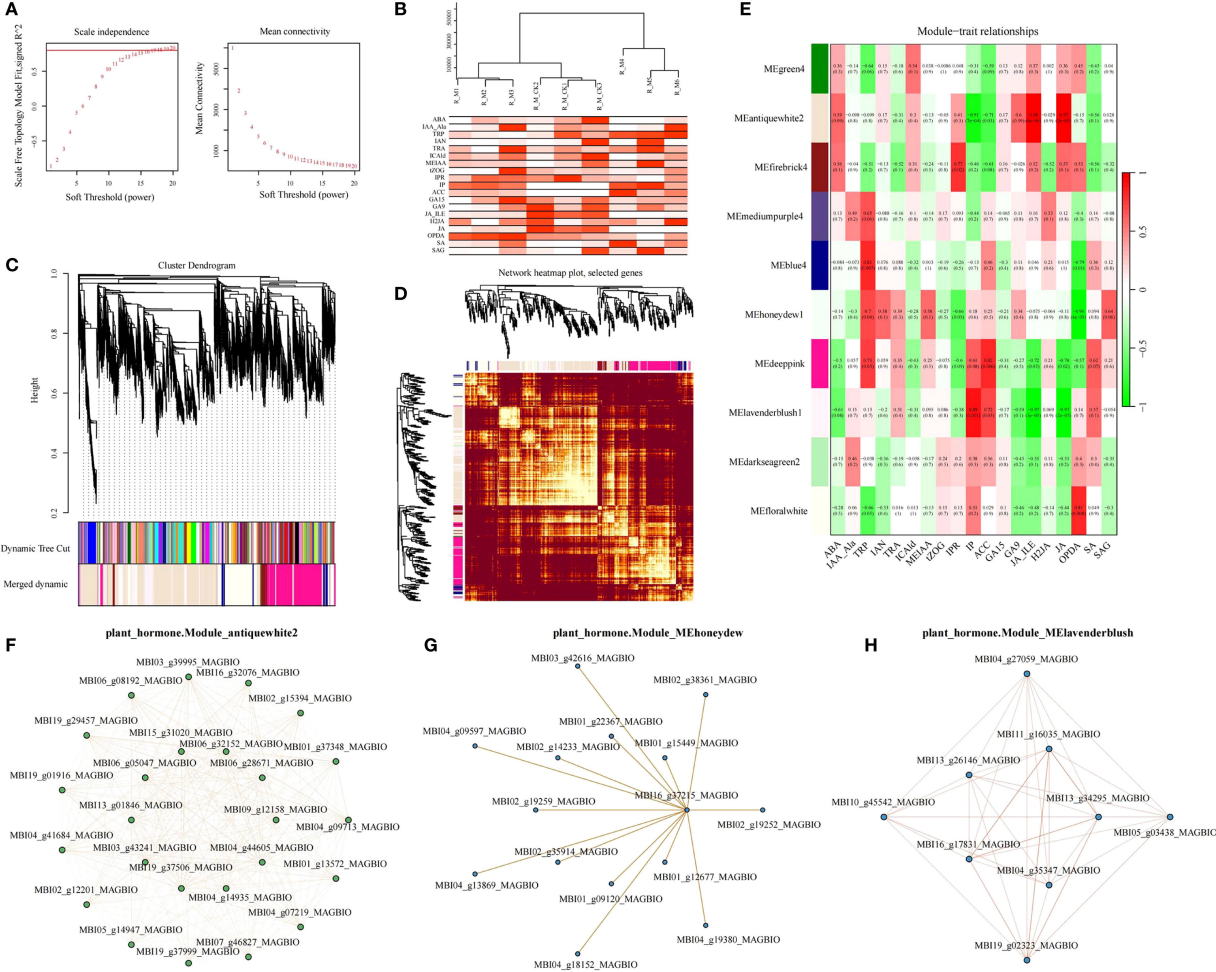
WGCNA analysis and correlation networks of hub genes in 3 modules. **(A)** WGCNA analysis soft threshold identification. **(B)** sample cluster tree and trait abundance map. **(C)** module cluster tree map. **(D)** module gene cluster heat map **(E)** module and trait correlation heat map. **(F)** Correlation networks of hub genes in Module_antiquewhite2. **(G)** Correlation networks of hub genes in Module_MEhoneydew1. **(H)** Correlation networks of hub genes in Module_Lavenderblusblush1.

**Figure 7:**
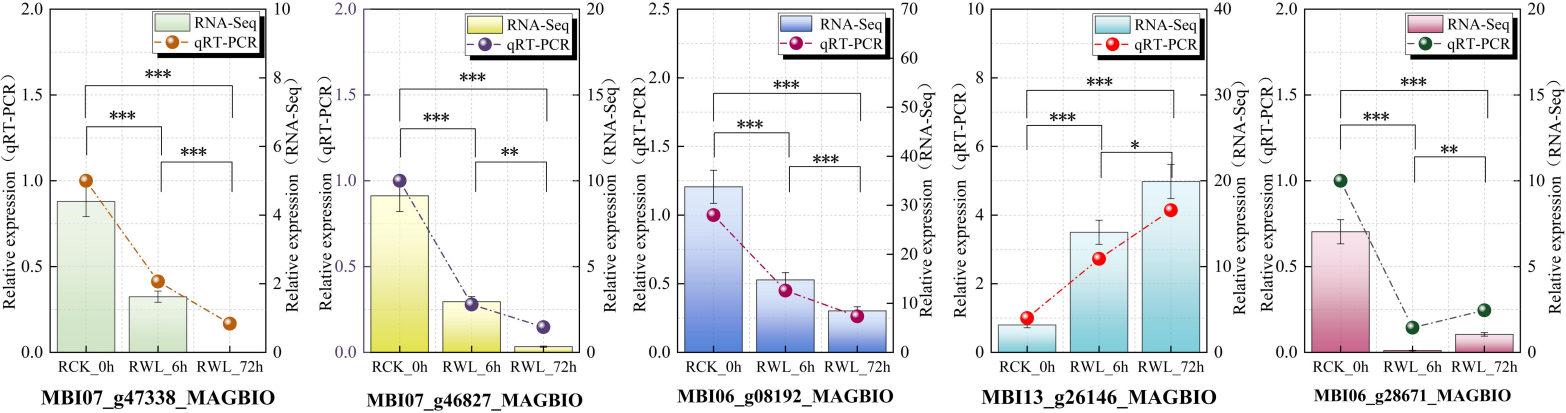
Expression verification of 5 candidate hub genes involved in plant hormone signaling transduction pathway under waterlogging stress. Differential expression was visualized through colored bars (qRT-PCR) and connected scatter points (RNA-Seq). All data are the means ± SE of three biological replicates, with the significance of intergroup differences indicated by asterisks above: *(P < 0.05), **(P < 0.01), ***(P < 0.001).

Building upon our finding that roots serve as the primary response organ to waterlogging stress in *M. sinostellata*, we employed WGCNA to elucidate the relationship between root hormone dynamics and transcriptional regulation. Using the WGCNA package (v1.72, R Core Team) with an optimal soft threshold power of 18, we identified 10 distinct gene modules (**Figures 6A, B**), each representing unique co-expression patterns. (**Figures 6A, B**). These gene modules are color-coded and represented in the form of cluster maps and network heat maps (**Figures 6C, D**). According to p<0.05&|R|>0.85, MEantiquewhite2 module was strongly correlated with phenotype IP (N6-isopentenadenine belongs to cytotinin), JA_ILE and JA (jasmonic acid), which were 0.89, -0.97 and -0.97, respectively (p<0.05). There was a significant and strong correlation between MElavenderblush module and phenotype IP, JA_ILE and JA (−0.91, 0.98, 0.97, respectively) (p<0.05). MEhoneydew module 12-oxophytodienoic acid (OPDA) (Jasmonic acid) had a strong negative correlation of 0.96 (p<0.05). In order to further search for candidate hub genes with important contributions in the gene network, we extracted annotation information of all these genes from the *Magnolia* gene annotation database. By comparison and integration of DEGs and annotation information, 71 genes related to plant hormone signal transduction pathway in 3 modules were selected as key candidate genes. Antiquewhite2 contained 61 hubgenes, honeydew1 contained 61 hubgenes, and Lavenderblusblush1 contained 9 hubgenes (**Supplementary Table S11**). The co-expression network of genes related to plant hormone signaling pathways in the three modules is shown in **Figure 7**. These hub genes were likely to be the key genes regulating the waterlogging tolerance of *M. sinostellata*.

Based on expression profiling of hormone-related genes, we identified five candidate hub genes and analyzed their transcriptional dynamics under waterlogging stress in *M. sinostellata* using qRT-PCR. The selected genes included: cytokinin dehydrogenase (MBI06_g28671_MAGBIO, MBI13_g26146_MAGBIO) and jasmonic acid-amino synthetase (MBI07_g47338_MAGBIO, MBI07_g46827_MAGBIO, MBI06_g08192_MAGBIO). Consistent with our Illumina HiSeq sequencing data, qRT-PCR analysis (**Figure 7**) revealed significantly changes of all five genes in *M. sinostellata* during waterlogging stress. These results suggest these hormone-related genes may play crucial regulatory roles in *M. sinostellata* waterlogging tolerance. The strong correlation between qRT-PCR and RNA-seq data further confirms the reliability of our transcriptome analysis.

### 3.5 The plant hormone signaling transduction of *M. sinostellata* in response to waterlogging stress

**Figure 8:**
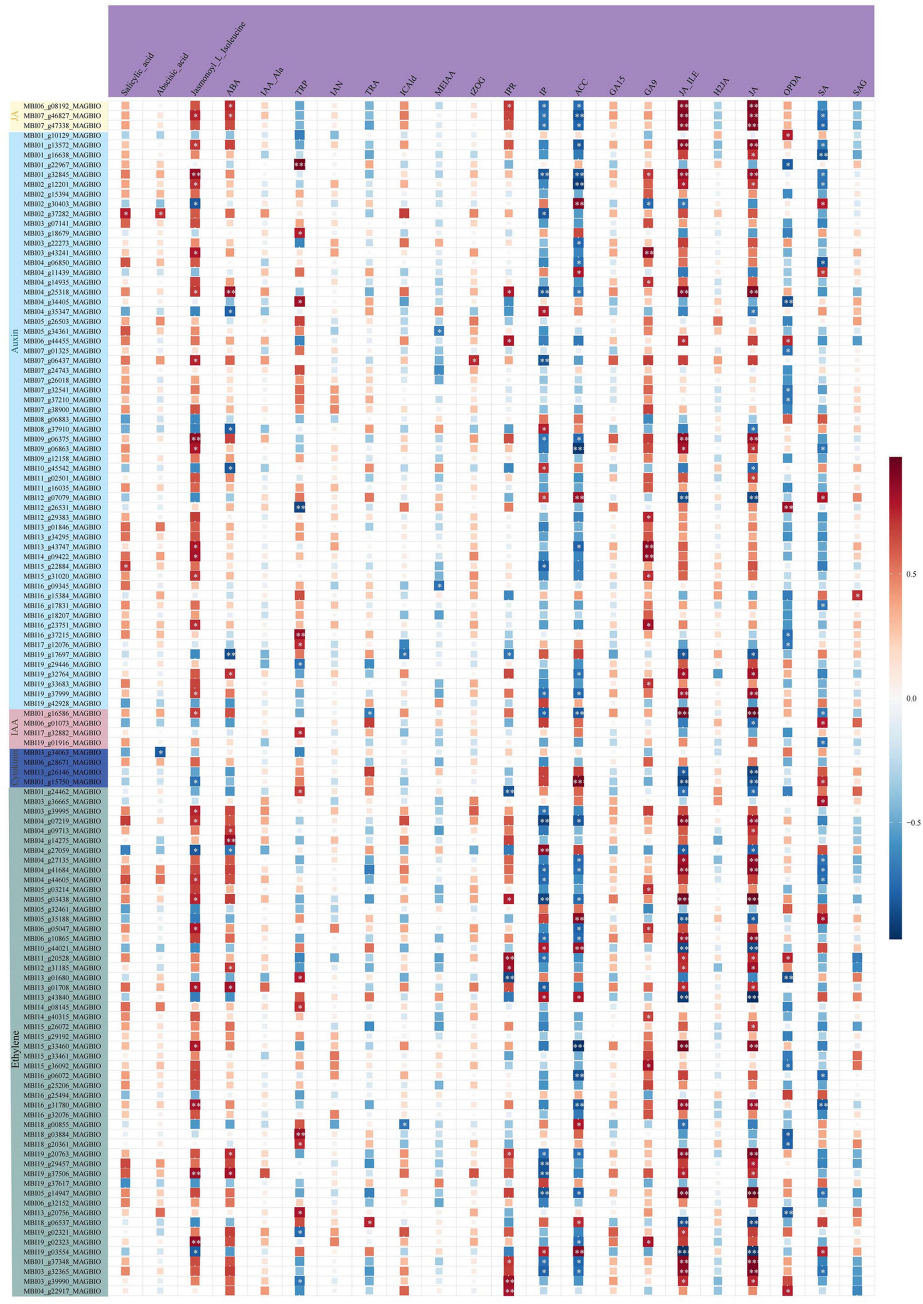
A heat map of the correlation between hormone-related DEGs and DEMs. In the heat map, the horizontal axis represents metabolites and the vertical axis represents genes. Red indicates a high positive correlation, while blue indicates a negative correlation. The darker the color, the stronger the correlation. The asterisk represents the degree of significance, ***(*p* < 0.0001), **(*p* < 0.01), and *(*p* < 0.05).

By combining transcriptomic and metabolomic results, we identified plant hormone signaling transduction as the important pathway of *M. sinostellata* in response to waterlogging stress. We analyzed the relationship between the selected key genes and metabolites by Spearman correlation analysis, and the results showed that all genes associated were changed (**Figure 5**). The pathway of plant hormone signal transduction was found to yield 102 DEGs and 22 DEMs, including genes related to IAA, CTK, GA, ET, ABA. The specific information is in the **Supplementary Materials** (**Supplementary Tables S10, S12**). All genes and metabolites in the JA signaling pathway were suppressed by waterlogging stress. Most genes were down-regulated in IAA signaling pathway, while almost all metabolites were accumulated in tryptophan metabolism. The GA signaling pathway related genes and metabolites were down-regulated in diterpenoid biosynthesis. In addition, ET signaling genes also changed (28 down-regulated and 14 up-regulated). Moreover, the metabolite ET was significantly accumulated after waterlogging stress. In addition, we selected all DEMs and DEGs for correlation analysis to determine the correlation between differential metabolites and differential expression genes in the plant hormone signaling transduction pathway (**Figure 8**). The results showed that many genes in this pathway were highly correlated with metabolites. In conclusion, waterlogging stress notably influenced the gene expression and levels of hormones such as IAA, CTK, GA, JA and ET in the roots of *M. sinostellata.*

## 4 Discussion

In recent years, accelerating climate change has led to increased frequency and intensity of waterlogging events worldwide (Li et al., 2022). Consequently, waterlogging stress has emerged as a major constraint limiting the cultivation and landscape application of ornamental plants (Voesenek and Bailey-Serres, 2015). The widespread horticultural use of *Magnolia* species, renowned for their exceptional ornamental value, is significantly constrained by their susceptibility to waterlogging damage (Wang et al., 2022). Therefore, identifying novel genes and metabolites to enhance their tolerance mechanisms is critical. In this study, we employed the waterlogging-tolerant species *M. sinostellata* as a model to characterize transcriptomic and metabolomic responses during waterlogging stress.

### 4.1 Morphological and anatomical changes under waterlogging stress

Waterlogging triggers a coordinated escape response comprising four synergistic adaptations: lenticel hypertrophy (improving O_2_ uptake), adventitious roots formation (compensating for root hypoxia), aerenchyma production (creating internal air channels), and internode elongation regulation (facilitating aerial emergence) (Pedersen et al., 2021). The formation of adventitious roots during waterlogging stress facilitates gas exchange and nutrition absorption (Steffens and Rasmussen, 2016). To a greater extent, these root tissues usually replace primary roots that die as a result of hypoxic stress, allowing normal growth and development to progress (Eysholdt-Derzsó and Sauter, 2017; Li et al., 2022). The development of aerenchyma, which facilitates oxygen diffusion to the root tips, is a well-documented response to hypoxia caused by waterlogging (Shiono et al., 2014; Yamauchi et al., 2018). The presence of hypertrophic lenticels is thought to facilitate gas exchange (Herzog et al., 2016). Under waterlogging stress, *M. sinostellata exhibited* significant morphological and anatomical adaptations, including hypertrophic lenticels, adventitious roots, and aerenchyma formation (**Figures 1B, C**). These morphological and anatomical changes are consistent with findings in other waterlogging-tolerant species, such as *Populus deltoides* and *Cucumis sativus*. These findings align with the broader understanding that waterlogging-tolerant plants often exhibit morphological adaptations to improve oxygen availability in submerged tissues (Colmer and Voesenek, 2009).

### 4.2 Integrated transcriptomic and metabolomic analysis

The integration of transcriptomic and metabolomic data revealed a strong correlation between the expression of genes involved in plant hormone signaling and the accumulation of corresponding metabolites (**Figures 6**, **5**). This finding is consistent with recent studies in *Hordeum vulgare* and *Triticum aestivum*, where the coordination of gene expression and metabolite accumulation is crucial for waterlogging tolerance (Wang et al., 2024). For the screening of key candidate genes, this study used the WGCNA method, which has been reported in many studies (Tian et al., 2024; Niu et al., 2024). In our study, the co-expression network analysis identified several key hub genes involved in plant hormone signaling (**Figure 6**). These genes were highly correlated with the accumulation of specific metabolites, such as jasmonic acid and CTK, suggesting that they play a crucial role in the response of *M. sinostellata* to waterlogging stress. The identification of these hub genes provides potential targets for future breeding and genetic engineering efforts aimed at improving waterlogging tolerance in ornamental plants.

### 4.3 Extensive alteration of genes involved in plant hormone signaling transduction pathway under waterlogging stress

Plant hormones function as master endogenous regulators that orchestrate multifaceted signaling networks by integrating interconnected hormonal cascades during waterlogging stress (Habibi et al., 2023; Rajesh et al., 2022). Our study identified significant changes in hormone-related genes and metabolites, revealing a complex interplay of hormonal responses that facilitate the adaptation of *M. sinostellata* to waterlogged conditions.

CTKs serve as pivotal regulators that help plants resist waterlogging stress. Islam et al. suggested that CTKs could be used for managing waterlogging-induced damage to mungbean (Islam et al., 2021). Cytokinin Oxidase (CKX) is a key enzyme that catalyzes the irreversible degradation of active CTK, thereby negatively regulating endogenous CTK levels (Jameson and Song, 2016; Kieber and Schaller, 2018). CTK homeostasis is tightly regulated by a suite of metabolic enzymes, including those involved in biosynthesis (Isopentenyltransferase, IPT), activation (Lonely Guy, LOG), degradation (CKX), reversible inactivation (zeatin O-glucosyltransferases, ZOGs), reactivation (β-glucosidases, GLUs), and irreversible N-glycosylation (UDP glycosyltransferases, UGTs). The cytokinin oxidase isoforms IP and IPR catalyze the oxidation of CTKs into inactive forms. Following their synthesis in plants, these enzymes modulate CTK activity, thereby regulating plant growth and developmental processes (Werner and Schmülling, 2009). In our study, the of *CKX* genes were down-regulated, while the CTK metabolites up-regulated (such as trans-Zeatin O-Glucosyltransferase, (tZOG)) (**Figure 5**), suggest that the plant reduces CTK degradation and increases active CTK levels to promote cell division and adventitious roots development, thereby enhancing adaptation to hypoxia.

JA plays a pivotal role in mediating plant responses to waterlogging stress (Yuan et al., 2017). Increasing the JA level through mutations or exogenous JA application has been shown to lead to inhibition of root growth in *Arabidopsis thaliana*, rice and cucumber (Sanders et al., 2000; Liu et al., 2015; Xu et al., 2016; Pan et al., 2025), which is tempting to speculate that JA is an inhibitor of adventitious rooting. The jasmonate-amido synthetase jasmonate resistant 1 (JAR1) plays a critical role in JA signaling by catalyzing the biosynthesis of jasmonyl-L-isoleucine (JA-Ile), the bioactive JA conjugate, from jasmonic acid (Staswick, 2008). Our research reveals the jasmonic acid (JA) signaling pathway is significantly suppressed after waterlogging stress, as evidenced by the down-regulation of *JAR1*, JA and its precursor 12-oxophytodienoic acid (OPDA), as well as the reduction in JA-Ile levels. Concurrently, the increase in hydroxylated JA (H2JA) further reduces JA activity (**Figure 5**). Our results are consistent with the studies on the response of cucumber and *A.* thaliana to waterlogging stress, suggesting that jasmonic acid plays a negative regulatory role in the formation of adventitious roots. Since this is inconsistent with the regulation of JA to waterlogging stress by other plant species (Arbona and Gomez-Cadenas, 2008; Xu et al., 2016; Ateeq et al., 2025), the specific mechanism remains to be further studied.

Our results also showed that the expression of genes encoding signaling components of other major plant hormones were changed under waterlogging stress, such as IAA, GA and ET. Emerging evidence reveals that both IAA transport and signal transduction pathways are integral to waterlogging stress adaptation, orchestrating physiological and morphological adjustments in oxygen-deprived environments (Kazan, 2013; Sharif et al., 2022). IAA can induce root apical meristematic tissue, which is an important factor regulating the formation of adventitious roots under waterlogging stress (Agulló-Antón et al., 2014). In our study, key genes in the IAA signaling pathway, such as *AUX1*, *IAA*, *GH3*, *ARF*, and *SAUR*, are down-regulated, which probably suppresses primary root elongation and redirects IAA flux to stem tissues, promoting the formation of adventitious roots. The up-regulation of IAA precursors, including methyl indole-3-acetic acid (MEIAA), tryptophan (TRP), tryptamine (TRA), and indole-3-acetyl-alanine (IAA-Ala), coupled with the down-regulation of the IAA catabolite ICAld, indicates that the plant accumulates IAA precursors and suppresses degradation to maintain IAA homeostasis, enabling rapid responses to environmental changes. GA plays a pivotal role in mitigating abiotic stresses induced-perturbations in plants by modulating various physio-biochemical and molecular processes. Under waterlogging stress, wheat exhibited up-regulated expression of GA metabolic genes (*GA3ox2* and *GA2ox8*) to promote the formation of adventitious roots (Nguyen et al., 2018); GA levels were significantly elevated in tolerant variety HX of peach (Ateeq et al., 2025). Besides, waterlogging experiments revealed that *GID1* mutation in rice, which encodes a soluble GA receptor, suppressed chlorophyll breakdown and accelerated carbohydrate metabolic turnover. This demonstrates that *GID1*-dependent GA signaling plays a pivotal role in plant waterlogging tolerance by fine-tuning carbohydrate utilization under hypoxic stress (Sun, 2010). However, in our research, *GA20o*x and *GID1* were down-regulated, which may inhibit energy-intensive growth, thereby enhancing hypoxia tolerance. ET serves as a pivotal regulator orchestrating both physiological and morphological adaptations in plants under waterlogging stress (Kuroha et al., 2018; Shen et al., 2022). Consistent with other studies, the ET signaling pathway in *M. sinostellata* is also activated, with the up-regulation of ethylene receptor (*ETR*), ethylene insensitive (*EIN*), and ethylene response factor 1 (*ERF1*) genes promoting the formation of aerenchyma and adventitious roots, which presumably improve oxygen transport and root functionality under hypoxic conditions (Chen et al., 2024; Li, 2021).

In summary, the above research results indicate that suggesting that IAA, CTK, JA, GA, ET signaling are positively involved in regulating the tolerance of *M. sinostellata* to waterlogging stress, although the precise molecular mechanisms require further elucidation.

## 5 Conclusion

In conclusion, this study provides the first comprehensive molecular characterization of waterlogging tolerance in *M. sinostellata* through integrated multi-omics analyses, revealing JA as a negative regulator, contrasting with its positive role in other species. Furthermore, we also indentified some potential candidate genes (*CKX* and *JAR1*) and metabolic markers (OPDA, JA-Ile, tZOG) that orchestrate coordinated morphological, physiological and molecular adaptations. The identified hub genes and metabolic markers offer valuable resources for molecular breeding of waterlogging-resistant ornamental trees, addressing a critical need in urban landscaping under climate change scenarios. To advance breeding efforts, subsequent work should focus on experimentally validating critical genes and hormonal pathways, mapping their regulatory crosstalk, and identifying trait-specific adaptations that improve the resilience to waterlogging stress in *Magnolia*.

## Data Availability

The original contributions presented in the study are publicly available. This data can be found here: https://www.ncbi.nlm.nih.gov/, accession number PRJNA1274043.
